# Advances in the role of STAT3 in macrophage polarization

**DOI:** 10.3389/fimmu.2023.1160719

**Published:** 2023-04-04

**Authors:** Tingting Xia, Meng Zhang, Wei Lei, Ruilin Yang, Shengping Fu, Zhenhai Fan, Ying Yang, Tao Zhang

**Affiliations:** ^1^ Key Laboratory of Cell Engineering of Guizhou Province, Affiliated Hospital of Zunyi Medical University, Zunyi, Guizhou, China; ^2^ Department of Dermatology, Affiliated Hospital of Zunyi Medical University, Zunyi, Guizhou, China; ^3^ The Clinical Stem Cell Research Institute, Affiliated Hospital of Zunyi Medical University, Zunyi, Guizhou, China

**Keywords:** STAT3, M1 macrophage, M2 macrophage, signaling pathway, macrophage polarization

## Abstract

The physiological processes of cell growth, proliferation, differentiation, and apoptosis are closely related to STAT3, and it has been demonstrated that aberrant STAT3 expression has an impact on the onset and progression of a number of inflammatory immunological disorders, fibrotic diseases, and malignancies. In order to produce the necessary biological effects, macrophages (M0) can be polarized into pro-inflammatory (M1) and anti-inflammatory (M2) types in response to various microenvironmental stimuli. STAT3 signaling is involved in macrophage polarization, and the research of the effect of STAT3 on macrophage polarization has gained attention in recent years. In order to provide references for the treatment and investigation of disorders related to macrophage polarization, this review compiles the pertinent signaling pathways associated with STAT3 and macrophage polarization from many fundamental studies.

## 1 Introduction

The signal transducer and activator of transcription (STAT) family, which is widely distributed in various mammalian tissues and cells, is a group of transcription factors that are activated by cytokines, growth factors, and other peptide ligands, and the role of STAT is to activate the transcription of target genes by binding to the promoters of target genes and sending signals from the cell membrane to the nucleus (1). Almost all types of mammalian cells have STAT3, a member of the STAT family that has undergone the most extensive research, in their cytoplasm. As an acute-phase response factorin interleukin (IL)-6 signaling, STAT3 was first discovered in 1994 and has similar functional domains to other STAT family members (2). Numerous studies have shown that aberrant or persistent activation of STAT3 has anti-inflammatory or pro-inflammatory, pro-fibrotic or anti-fibrotic (3), and oncogenic effects, and aberrantly expressed STAT3 has also been shown to be present in a variety of inflammatory and immune diseases, fibrotic diseases, and tumors (4).

By phagocytosing of pathogens, presenting exogenous antigens, and secreting cytokines and growth factors, macrophages play a crucial part in the physiopathological processes of immune defense, inflammatory response, tissue remodeling, and homeostasis of the body (5). According to their immunological functional distinctions, polarized macrophages can be divided into M1-type macrophages of the classic activation pathway and M2-type macrophages of the alternative activation pathway (6). Transcription factors are key determinants of macrophage polarization and are closely linked to various other signaling molecules. These transcription factors include STATs, interferon-regulatory factor, nuclear factor kappaB (NF-κB), activator protein 1, peroxisome proliferator-activated receptor-γ, Krüppel-like factor 4 etc (7, 8). Activation of these key transcription factors controls macrophage skewing toward the M1 phenotype or toward the M2 phenotype. Among them, STAT3, a member of the STATs family, has been shown to be involved in macrophage polarization and plays an important role in a variety of inflammatory-immune, fibrotic and neoplastic diseases (9–11). Therefore, this review used “STAT3”, “macrophage polarization” and “signaling pathway” as search terms and searched PubMed, Web of Science and Google Scholar to summarize the relevant signaling pathways and their regulatory mechanisms of STAT3-regulated macrophage polarization, hoping to provide direction and basis for the mechanism research and therapeutic strategy development of related diseases involving STAT3 and macrophage polarization.

## 2 STAT3’s structural and functional characteristics

The STAT family consists of seven structurally similar and highly conserved members: STAT1, STAT2, STAT3, STAT4, STAT5A, STAT5B and STAT6 (12), all of them containing six conserved structural domains, namely: (i) N-terminus domain (NTD), which is involved in STAT3 dimer nuclear translocation and cooperative DNA binding; (ii) Coiled-coil domain (CCD), which is the functional domain of STAT interacting with other transcription factors and co-activators; (iii) DNA-binding domain (DBD), which mediates recognition and binding of particular DNA; (iv) Linker domain (LD), which performs a linking function and keeps DBD structurally stable; (v) Src homology region 2 (SH2) domain, which is highly conserved and mediates protein-protein interactions and promotes STAT dimer formation; (vi) C-terminal transactivation domain (TAD), which is a phosphorylation region of STAT and enhances transcriptional activity (13, 14).

The gene encoding STAT3 is located on chromosome 17 (17q21) and consists of 750 to 795 amino acids, with a protein molecular mass of approximately 89 kDa (15). As a downstream intracellular effector of inflammatory and growth factors, STAT3 is widely expressed in various cells and tissues. At the microscopic level, it regulates cell proliferation, migration, apoptosis, and other fundamental processes; at the macroscopic level, it is linked to the development of mammalian individuals (16). STAT3 is activated by phosphorylation, a process that is very tightly controlled in normal tissues and of short duration. It has been documented that the phosphorylation level of STAT3 peaks 15-60 min after stimulation by cytokines and other factors, and then gradually decreases (17). The activation of STAT3 is closely correlated with the phosphorylation of tyrosine 705 and serine 727 of TAD, and activated STAT3 forms a dimer into the nucleus to perform functions in signaling transduction and transcriptional activation (18).

Four different STAT3 isoforms, STAT3α (92 kDa), STAT3β (83 kDa), STAT3γ (72 kDa) and STAT3δ (64 kDa), are regarded to be important factors that influence STAT3 functional heterogeneity (19). Among them, STAT3α, the most common isoform of STAT3, is generated by transcription and translation of the full-length STAT3 gene, which contains two phosphorylation sites of tyrosine and serine in the TAD region and can regulate the cellular response of macrophages to IL-6 and IL-10, and is involved in important biological processes such as immune regulation, cell proliferation and differentiation, and tumor cell proliferation and migration (20). Compared with STAT3α, STAT3β lacks 55 C-terminal amino acid residues and contains only tyrosine phosphorylation sites in the TAD region, which has the function of negative regulation of transcription, and STAT3β has better specific DNA binding activity and is involved in regulating inflammatory factors, tumor microenvironment and immune cells, inhibiting tumor infiltration and progression, and reducing chemotherapy resistance (19, 21). STAT3γ and STAT3δ are derived from the proteolytic processing of STAT3 and are activated mainly at various stages of granulocyte differentiation (22, 23), but there are few studies on both.

The distinct structures of STAT3 determine the heterogeneity of its activity. However, there are relatively few studies on STAT3 isoforms, and if we focus our study attention on this, it may explain why STAT3 has varied biological roles under the influence of various local microenvironments.

## 3 Phenotypes and functions of macrophage polarization

Macrophages are derived from monocytes that originate from hematopoietic stem cells in the bone marrow. Monocytes grow and mature in the bone marrow for up to 24 hours before moving through the bloodstream (24, 25). Prior research has demonstrated that circulating monocytes can differentiate into macrophages in response to damage or inflammation or can migrate into tissues and become resident macrophages (26). Recent research has revealed that whereas some subpopulations of tissue-resident macrophages are descended from adult circulating monocytes, the majority of tissue-resident macrophages are produced from the yolk sac during embryonic development (27).

**Figure 1:**
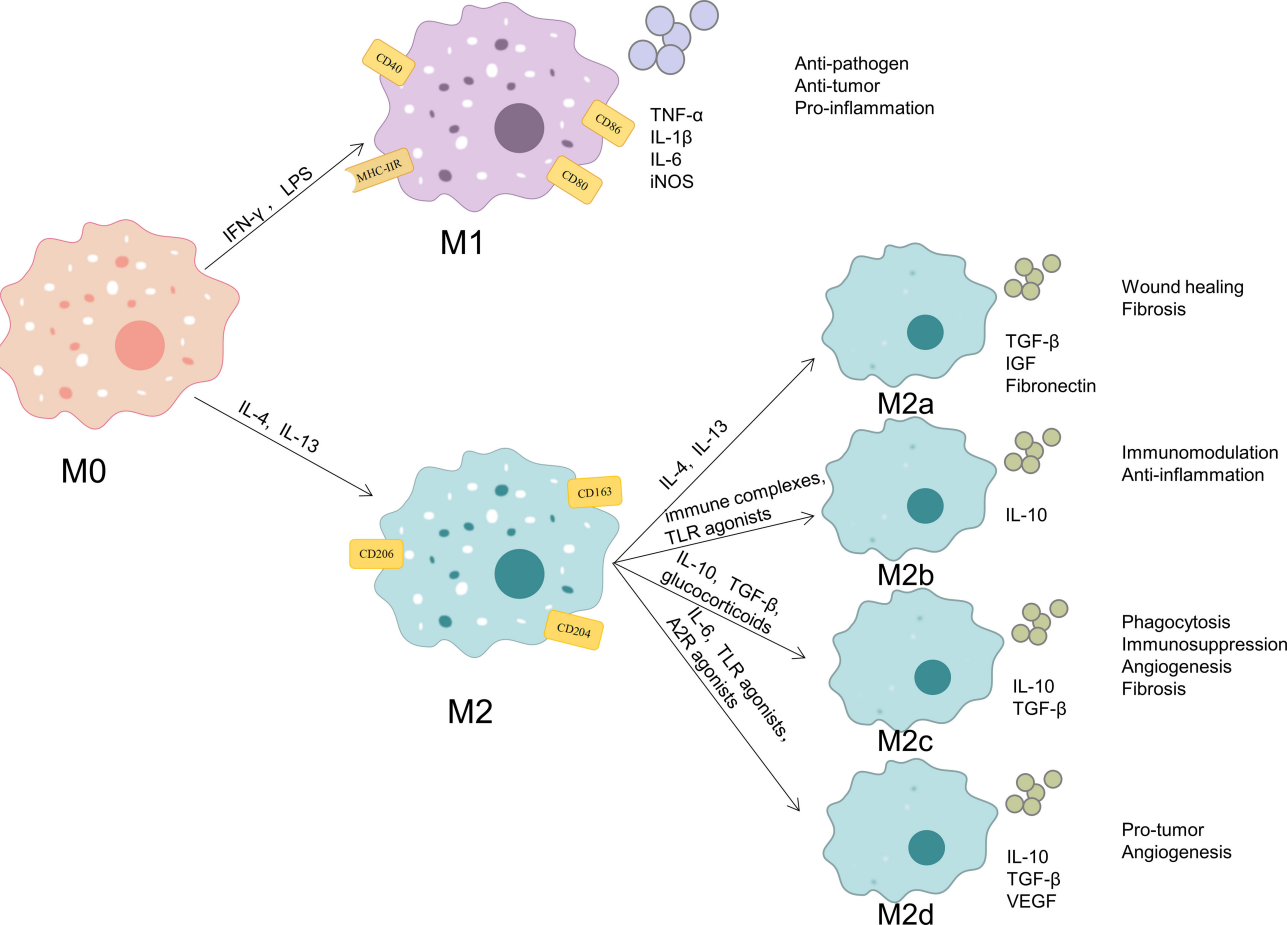
Phenotypes and functions of macrophage polarization. When exposed to different microenvironmental stimuli, M0 macrophages can be polarized into M1 macrophages and M2 macrophages. M2 macrophages can be subdivided into M2a, M2b, M2c and M2d. M1-type macrophages express the cell surface markers CD40, CD80, CD86, and MHC-IIR and release pro-inflammatory molecules such as TNF-α, IL-1β, IL-6, and iNOS. These cells possess strong anti-pathogen, anti-tumor, and pro-inflammatory effects. M2-type macrophages express the cell surface markers CD163, CD204 and CD206. M2a macrophages secrete like TGF-β, IGF, and fibronectin, and they can promote wound healing and fibrosis; M2b macrophages secrete IL-10, and they have strong immunomodulatory and anti-inflammatory effects;M2c macrophages secrete TGF-β and IL-10, and they exert phagocytosis, immunosuppression, angiogenesis, and development of tissue fibrosis;M2d macrophages secrete IL-10, TGF-β, and VEGF, and they promote angiogenesis and cancer metastasis.

Macrophages are quite heterogeneous and depending on the tissue they are found in have different names, which including microglia (found in the central nervous system), osteoclasts (found in the bone), Kupffer cells (found in the liver), alveolar macrophages (found in the lung), histiocytes (found in the spleen and connective tissue), intestinal tissue macrophages, and Langerhans cells (found in the skin). Resident macrophages promote tissue homeostasis and monocyte-derived macrophages mainly assist in host defense (28). The ability of macrophages to modify their phenotype and function in response to changes in the local microenvironment, named macrophage polarization, is another characteristic of macrophages (**Figure 1**). Macrophages can be classified into two phenotypes: the classic activated M1-type macrophages and the alternatively activated M2-type macrophages, depending on the surface receptor expression and secretion profile. Interferon (IFN)-γ and bacterial lipopolysaccharide (LPS) are typically used to stimulate M1-type macrophages, which release pro-inflammatory molecules such as tumor necrosis factor (TNF)-α, IL-1β, IL-6, and nitric oxide synthase (iNOS) as well as secretes a number of chemokines. Additionally, these macrophages express the cell surface markers CD40, CD80, CD86, and major histocompatibility complex class II receptor. M2-type macrophages are induced by IL-4 or IL-13 and secrete IL-10, transforming growth factor (TGF)-β, insulin-like growth factor 1, vascular endothelial growth factor (VEGF), epidermal growth factor (EGF), platelet-derived growth factor (PDGF), and other cytokines, as well as increased cell surface expression of Arginase (Arg)1 and the cell surface markers CD163, CD204 and mannose receptor (CD206) (5, 29, 30). Therefore, the secretion profile of macrophages and associated markers can be used to identify the polarization status of macrophages. Functionally, M1-type macrophages have potent anti-pathogen and anti-tumor activities, promote inflammatory responses in the early stages of inflammation, kill intracellularly infected pathogens, mediate reactive oxygen species-induced tissue damage, and are detrimental to tissue regeneration and wound healing; M2-type macrophages, also known as reparative macrophages, have potent phagocytosis, remove debris and apoptotic cells, promote tissue repair and wound healing, and have pro-angiogenic and pro-fibrotic properties (31–33).

According to various stimulating factors, M2 macrophages can be further separated into the four subtypes M2a, M2b, M2c, and M2d. M2a macrophages are stimulated by IL-4 or IL-13 to generate and secrete pro-fibrotic substances like TGF-β, insulin-like growth factor, and fibronectin in order to promote tissue repair; M2b macrophages are triggered by immune complexes and Toll-like receptor (TLR) agonists and produce high amounts of the anti-inflammatory cytokine IL-10, which has strong immunomodulatory and anti-inflammatory effects;M2c macrophages are triggered by IL-10, TGF-β, or glucocorticoids and exert phagocytosis and clearance of apoptotic cells, immunosuppression, stimulation of angiogenesis, and development of tissue fibrosis through high expression of Mer receptor tyrosine kinase and high levels of TGF-β and IL-10 release;M2d macrophages are triggered by IL-6, TLR and A2 adenosine receptor agonists and characterized by high expression of IL-10, TGF-β, and VEGF and low expression of IL-12, TNF-α, and IL-1β, which promotes angiogenesis and cancer metastasis (34–36).

Since various macrophage subpopulations have different secretion profiles, macrophages can regulate a variety of pathophysiological processes, including various inflammatory and immune diseases, fibrotic diseases, and tumors. This coincides with the effects of STAT3 signaling pathway, and numerous studies have also demonstrated that macrophages can act as STAT3 effectors, secreting different cytokines, chemokines and growth factors through macrophage polarization, which play important roles in tissue homeostasis, tissue remodeling, immune response and cancer progression. However, in actuality, macrophage polarization is a dynamic process, and the distinction between M1 and M2 macrophages is a simplified explanation of the complex process of polarization, which involves the balancing of various cytokines and chemokines as well as the recruitment of other immune cells or cancer cells (5). Moreover, although macrophages can be classified into the aforementioned types, there are very few studies concerning macrophage subtypes under physiological or pathological conditions on them. Therefore, this review focuses on studies involving M1 and M2 macrophage types.

## 4 Research on the signaling pathways related to STAT3 regulation of macrophage polarization

**Figure 2:**
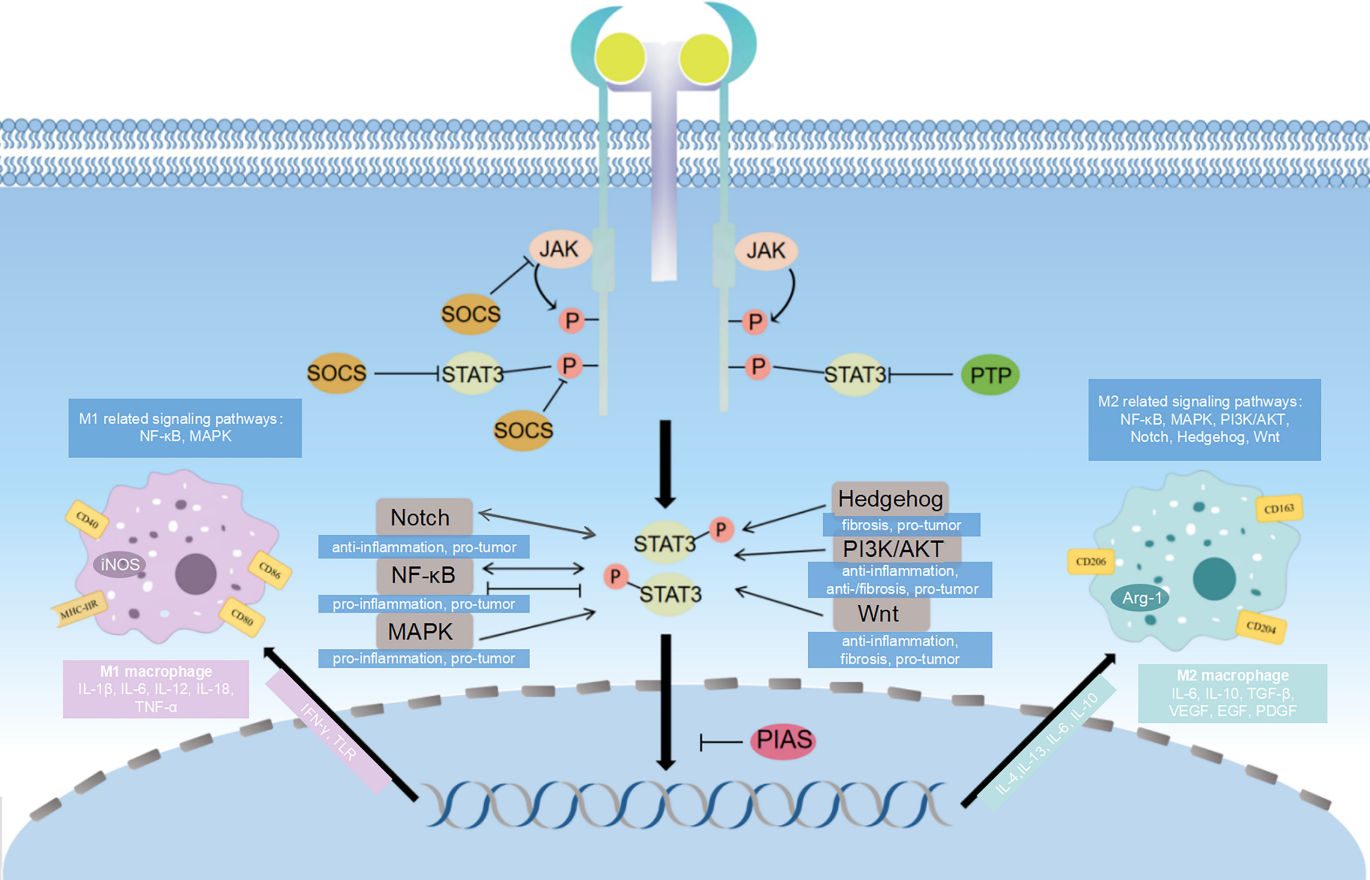
Diagram of signaling pathways related to STAT3 and macrophage polarization. STAT3 influence macrophage polarization through different signaling pathways. The major pathway associated with STAT3 is JAK/STAT signaling pathway, and SOCS, PIAS and PTP can inhibit different targets of this signaling pathway, which ultimately affects the direction of macrophage polarization. The NF-κB signaling pathway, PI3K/AKT signaling pathway, Notch signaling pathway, Hedgehog signaling pathway, Wnt signaling pathway and MAPK signaling pathway crosstalk with JAK/STAT3 signaling pathway, which regulates gene transcription and have effects on macrophage polarization.

Macrophage polarization is regulated through the activation of several interrelated cellular signaling pathways. STAT3 plays an extremely important role in macrophage polarization as a crossroads of multiple signaling pathways. The macrophage polarization-related pathways involving STAT3 include: Janus kinases (JAK)/STAT signaling pathway, NF-κB signaling pathway, phosphoinositide 3-kinase (PI3K)/protein kinase B (AKT) signaling pathway, Notch signaling pathway, Hedgehog (Hh) signaling pathway, Wnt signaling pathway, mitogen-activated protein kinase (MAPK) signaling pathway, etc (**Figure 2**).

### 4.1 STAT3 regulates macrophage polarization through the JAK/STAT signaling pathway

JAK/STAT is a main signaling pathway for the activation of more than 70 cytokines, and it plays an important role in numerous crucial biological processes such as cell proliferation, differentiation, apoptosis and immune regulation (37). JAK1, JAK2, JAK3, and tyrosine kinase (TYK) 2, which belong to non-receptor protein tyrosine kinases, are the four members of the JAK family. In the JAK/STAT signaling pathway, extracellular signals (mainly cytokines and growth factors) dimerize the receptors, which bring their coupled JAKs in close proximity to each other, and JAKs are activated by interactive tyrosine phosphorylation. And then the activated JAK in turn catalyzes the phosphorylation of tyrosine residues of the dimerized receptors to recruit STATs in the cytoplasm, which binds to the receptors and are phosphorylated by the activated JAK. Finally, the phosphorylated STATs separate from the receptors to form a dimer in the cytoplasm and translocate to the nucleus, where they bind to the promoter of the target gene to activate gene transcription (38).

STAT3 is a multifunctional protein that is regulated by various cytokines and growth factors, and influences human metabolism, immune inflammation and damage repair through JAK/STAT signaling (39). According to studies on the involvement of JAK/STAT3 in macrophage polarization, either inhibition or activation of this pathway increases macrophage M2 polarization in different diseases. In most studies, however, activation of STAT3 stimulates macrophage M2 polarization. According to one study, JAK2/STAT3 activity was significantly increased in rats with severe acute pancreatitis, and JAK2 inhibition increased levels of cytokines (such as Foxp3, IL-10, and TGF-β) necessary for M2 macrophage polarization, leading to a reduction in the secretion of pro-inflammatory factors IL-6, IFN-γ, and TNF-α, which prevents pancreatic inflammation and damage (40). In animal models of myocardial ischemia/reperfusion injury and psoriasis, it was also demonstrated that inhibition of the JAK/STAT3 signaling pathway promoted macrophage M2 polarization, which in turn reduced pro-inflammatory factors and upregulated anti-inflammatory and pro-fibrotic factor levels, thereby favorably alleviating tissue inflammation and inhibiting tissue damage (41, 42). While cucurbitacin B, a widely used triterpenoid compound, was able to decrease the phosphorylation of JAK2 and STAT3 and the nuclear translocation of p-STAT3, thereby limiting the migration of rectal cancer cells, it was also demonstrated *in vivo* that cucurbitacin B enhanced antitumor immunity through regulating M2 polarization of macrophages, but the specific mechanism of action by which macrophage polarization affects tumor progression was not described (43). Zeng et al. also found that activation of the JAK2/STAT3 signaling pathway promoted the polarization of M2 macrophages, which in turn decreased the levels of inflammatory factors IL-1β, TNF-α and IL-18 while increasing the levels of anti-inflammatory factor IL-10 significantly improved the skin lesions in mice with atopic dermatitis (44). Other studies, especially those related to tumor diseases such as ovarian cancer and glioma, have shown that activation of the JAK/STAT3 signaling axis facilitates M2 macrophage polarization and affects disease progression by activating or inhibiting related cytokines (45–49). The two completely opposite types of results imply that macrophage polarization is not only related to the heterogeneity of STAT3 and macrophages themselves, but also influenced by the JAK/STAT3 pathway, as well as multiple cytokines, chemokines, immune cells, tumor cells, and crosstalk of different signaling pathways in macrophages (50, 51). The balance of these factors may determine the direction of macrophage polarization.

The two most significant upstream cytokines of the JAK/STAT3 signaling pathway are IL-6 and IL-10. IL-6, which is widely present in the tumor microenvironment, is a key gene involved in promoting tumor cell cycle progression and inhibiting apoptosis (52). Overactivation of STAT3 signaling has also been observed in the majority of human malignancies and is linked to a poor prognosis (15). IL-6 activates the JAK/STAT3 signaling pathway largely by binding to the host cell surface receptor complex glycoprotein 130/IL-6R (53). Current studies have shown that IL-6/JAK/STAT3, in addition to being expressed directly in tumor cells and promoting tumor cell proliferation, differentiation and metastasis (54), can also appear in macrophages and indirectly affect disease development and progression through macrophage M2 polarization (46). Therefore, macrophage polarization to M2 type can be inhibited by inhibiting this signaling pathway, suppressing the expression of pro-tumor related cytokines such as IL-6, IL-10 and VEGF, and ultimately slowing down tumor disease progression (47, 55). Expression of pro-inflammatory mediators like cell surface receptors, chemokines, and cytokines is inhibited by the anti-inflammatory factor IL-10 (56). Additionally, research conducted by our team has demonstrated that co-culturing peripheral blood mesenchymal stem cells with macrophages can encourage the polarization of macrophages toward M2 type *via* the IL-10/JAK/STAT3 pathway, as well as reduce the expression and secretion of pro-inflammatory factors while increasing the expression and secretion of anti-inflammatory ones (57). In studies of enterohepatic crosstalk in nonalcoholic steatohepatitis, it has also been demonstrated that activation of this pathway promotes M2-type macrophage polarization, which ultimately attenuates the intensity of inflammation in intestinal tissues and maintains intestinal homeostasis (58). Thus, stimulation of the IL-10/JAK/STAT3 signaling pathway may diminish or eliminate inflammation by boosting M2 macrophage polarization, which promotes regenerative tissue repair (59).

In order to convey extracellular signals to the nucleus and trigger gene transcription, STATs primarily receive signals from cytokines or growth factor/JAKs pathways, as was already mentioned. STAT3, as a member of the STATs family, has a highly complex regulatory bioactivity and can exert pro-tumor, pro-inflammatory or anti-inflammatory and tissue repair effects through the activation of ligands such as IL-6/IL-10. This effect is closely related to the polarization of macrophages, and by exploring its relationship with macrophage polarization, the pathogenic mechanisms of various inflammatory immune, fibrotic, tumorigenic and other related diseases can be initially explored and new and more targeted therapeutic approaches can be developed.

### 4.2 Effect of negative regulators of JAK/STAT3 signaling pathway on macrophage polarization

Activation of the JAK/STAT signaling pathway in normal cells is brief and quick, because several negative feedback loops are quickly activated and effectively block the STAT signaling pathway and the activity of downstream functional proteins (60). The most important negative regulators of JAK/STAT3 signaling pathway include suppressors of cytokine signaling (SOCS), protein inhibitors of activated stats (PIAS) and protein tyrosine phosphatases (PTP), which prevent overphosphorylation of STAT3 (61). The SOCS family is the most prevalent and significant inducible negative regulator among these inhibitors (62). Through primarily three mechanisms, SOCS negatively regulates the JAK/STAT signaling pathway (63, 64): (i) Preventing the transcription factor STAT from interacting with the cytoplasmic area of the cytokine receptor by competitively binding there with the SH2 structural domain, which is identical to STAT;(ii) Additionally, SOCS has the ability to attach to JAK *via* the SH2 structural domain and block it from binding to STAT by competing with it;(iii) Degradation of SOCS-bound signaling proteins such as JAK and STAT *via* the ubiquitination pathway, thereby inhibiting cytokine signaling. This negative regulation of SOCS assists the regulation of inflammatory immune responses and suppresses cell proliferation (65). Recent research has demonstrated that the SOCS protein family, particularly the SOCS3 protein, is crucial for macrophage polarization, specifically macrophage polarization of the M1 subtype (66–68). Therefore, macrophage polarization can be regulated by activating or inhibiting the SOCS3-regulated JAK/STAT3 pathway, providing new therapeutic targets for inflammation, fibrosis, tumor and other related diseases (69, 70). The second negative regulator, PIAS, is an endogenous inhibitor of STAT proteins, and PIAS3 inhibits STAT3 target gene transcription by blocking STAT3 dimer binding to DNA (71). Brassinosteroids, a plant antitoxin, were discovered to suppress PIAS3 and SOCS3 by inhibiting STAT3 signaling, which slowed tumor development (72). The third negative regulator, PTPs, inhibits downstream signaling by reducing STATs activity *via* p-STAT3 dephosphorylation (23, 73). At present, there are no studies on the triad of PIAS or PTP, STAT3 and macrophage polarization, but based on the available studies, it is not difficult to infer the relationship between the three. Therefore, we speculate that regulation of STAT3 and macrophage activation through inhibition of PIAS or PTP may become a focus of future research.

In summary, the negative regulators of the JAK/STAT3 signaling pathway, SOCS, PIAS and PTP, play different roles in the progression of related diseases by inhibiting signal transduction at different levels of the pathway and thus influencing the direction of macrophage polarization, but the relevant studies are insufficient, and further studies on the relationship among these negative regulators, STAT3 and macrophage polarization are needed to provide directions for the research and treatment of related diseases.

### 4.3 Other signaling pathways regulate macrophage polarization by affecting STAT3

It is generally understood that macrophage polarization is a complex process governed by several intracellular signaling molecules and their pathways, resulting in various activation states (74, 75). And STAT3 can regulate macrophage polarization to conduct many crucial roles in human normal and malignant tissues, such as differentiation, proliferation, survival, angiogenesis, and immune system regulation (76). In the STAT3-dependent macrophage polarization process, various signaling pathways build a complex network structure that interacts and influences one another to create signal crosstalk.

#### 4.3.1 NF-κB signaling pathway

The transcription factor NF-κB is the “master switch” that regulates the expression of different pro-inflammatory mediator genes. The p65 subunit of NF-κB regulates macrophage M1 polarization. The classical NF-κB pathway is activated when the Toll-like receptor 4 (TLR4) on the surface of macrophages binds to LPS through a pathway dependent on the myeloid differentiation factor 88 (MyD88) or the interferon regulatory factor (IRF) 3. This pathway includes IkappaB kinase (IKK) phosphorylation, degradation of IkappaBalpha (IκBα) after phosphorylation modification, and entry of p65/p50 of NF-κB into the nucleus to mediate transcription of pro-inflammatory factors such as IL-1β, IL-6 and TNF-α, amplifying inflammatory signals (77). In general, NF-κB activation causes an inflammatory macrophage M1 phenotype, and STAT3 activation is crucial for anti-inflammatory M2 phenotype. Together, these two proteins maintain M1/M2 homeostasis and coordinate responses to various microenvironments (78). The TLR4 pathway is negatively regulated by (MicroRNA) let7b, which has been demonstrated to be the most important cellular mechanism involved in immune system regulation and inflammation (79). TLR4 and p65 expression levels in macrophages were significantly reduced after treatment with let7b, whereas p-STAT3 and p-AKT expression levels were significantly time-dependently upregulated, promoting macrophage differentiation to M2 type, and STAT3 activation was completely inhibited in the presence of let-7b inhibitor, suggesting that regulation of macrophage polarization can be mediated through TLR4/NF-κB/STAT3/AKT regulatory signaling, which in turn produces a large number of mediators that fine-tune the inflammatory response and promote wound healing in diabetic skin wounds (80). Although the TLR4-MyD88 complex activates IκBα, NF-κB and IRF3 and thus participates in macrophage M1 activation, it has also been demonstrated that this pathway is not only directly engaged in M2-associated CD163, CD204, and IL-10 gene production, but is also essential for the downregulation of M1 inflammatory cytokines. Further studies revealed that JAK2 and TYK2 were recruited to MyD88 to induce phosphorylation and activation of STAT3, promote M2-type macrophage polarization, and induced production of VEGF, bEGF and PDGF, which exhibited pro-angiogenic activity and tumorigenicity in a mouse model of pancreatic adenocarcinoma (81). It has also been shown that NF-κB inhibitors significantly block the phosphorylation of STAT3, which skews macrophage activation toward M1 (82). Similarly, STAT3 activation is critical for NF-κB translocation into the nucleus (83), and inhibition of STAT3 phosphorylation synergistically inhibits activation of the NF-κB pathway (84, 85). Animal studies conducted *in vitro* and *in vivo* revealed that Astragalus IV, a Chinese herbal medicine, may prevent hepatocellular carcinoma cells from proliferating, invading, and migrating by inhibiting the polarization of M2 macrophages and reducing the secretion of TGF-β through inhibiting the TLR4/NF-κB/STAT3 signaling pathway (86). The NF-κB signaling pathway is complicated, similarly to the JAK/STAT3 pathway, and activation of this pathway can both promote and inhibit M2 macrophage polarization. Furthermore, the interaction between STAT3 and the NF-κB pathway is not well understood, and the two have both promoted and inhibited each other in related research; hence, further investigation is required to answer these problems.

#### 4.3.2 PI3K/AKT signaling pathway

Recent research has revealed that the PI3K/AKT signaling pathway is essential for macrophage survival, proliferation, and migration (87). Dysregulation of this signaling pathway has been linked to a number of human diseases, including cancer, diabetes, cardiovascular disease, and neurological disorders (88–91). V-set immunoglobulin-domain-containing 4 is a membrane protein that belongs to the immunoglobulin superfamily of complement receptors. This kind of protein was discovered to decrease activation in pro-inflammatory macrophages by boosting the PI3K/AKT/STAT3 cascade, increasing pyruvate dehydrogenase kinase-2 expression, and decreasing pyruvate dehydrogenase function *via* phosphorylation (92). Although it is now widely acknowledged that M2 macrophages contribute to the pro-fibrotic response, few researchers have hypothesized that M1 macrophages cause fibrosis by overexpressing pro-inflammatory cytokines such IL-6, IL-1β, and TNF-α, while the role of M2 macrophages in fibrosis has not been established because they have various isoforms (93–95). Previous research suggests that IL-10 may play a preventive function in the prevention of chronic fibroproliferative disorders (96). Adipose-derived stem cells stimulate macrophage M2 polarization *via* PI3K/AKT/STAT3-dependent pathway, which suppresses fibroblast development into myofibroblasts *via* local IL-10 production, lowering extracellular matrix synthesis and, ultimately, cardiac fibrosis (97, 98). Type Iγ phosphatidylinositol phosphate kinase is a phosphatidylinositol kinase that produces phosphatidylinositol bisphosphate in cells and increases STAT3 phosphorylation by stimulating activation of the PI3K/AKT signaling pathway, which in turn promotes the C-C motif chemokine ligand 2 transcription to improve tumor-associated macrophage recruitment (99). Other studies have also demonstrated that activation of PI3K/AKT promotes STAT3 phosphorylation and nuclear translocation, which facilitates the polarization of macrophages toward M2 and exerts immunomodulatory and pro-cancer effects through the secretion of a large number of mediators (100, 101). Therefore, it is anticipated that this pathway will serve as a possible therapeutic target for a range of disorders.

#### 4.3.3 Notch signaling pathway

The Notch signaling pathway consists of several highly conserved surface receptors that are involved in cell proliferation and apoptosis, influencing the development of various biological organs and tissues (102). Instead of a secreted protein as a ligand, the receptor and ligand of this signaling pathway are membrane proteins, which mediate the activation effect after two cells come into close contact with one another. When the Notch ligand binds to the receptor (Notch 1-4), the Notch intracellular domain translocates to the nucleus and subsequently activates downstream target genes upon binding to transcription factors (103). In recent years, many studies have reported that targeting the Notch signaling pathway can regulate macrophage polarization (104, 105), and Notch signaling can positively activate the JAK/STAT3 pathway (106, 107). Notch4 was found to reduce the expression of pro-inflammatory cytokines (including IL-6 and IL-12) and co-stimulatory molecules (such as CD80 and CD86) in macrophages, while decreasing STAT1 activation and increasing STAT3 activation (108). Interestingly, in a study of breast cancer, STAT3 was shown to be an upstream regulator of Notch, and inhibition of this signaling pathway inhibited macrophage M2 polarization and thus breast cancer metastasis (109). Therefore, these results imply that the Notch/STAT3 signaling pathway may be important in understanding several disorders linked to macrophage polarization.

#### 4.3.4 Hedgehog signaling pathway

During numerous developmental stages in both vertebrates and invertebrates, the Hedgehog signaling pathway regulates cell proliferation, differentiation, and apoptosis; aberrant stimulation of this signaling pathway can result in tumorigenesis and progression (110). Sonic Hedgehog (Shh), Desert Hedgehog (Dhh), and Indian Hedgehog (Ihh), members of the Hh family of signaling proteins, bind to Patched-1 (PTCH1) receptors on neighboring target cells and release Smoothened (Smo) repression as a result, ultimately leading to activation and nuclear translocation of Gli transcription factor members and upregulation of Hh target genes (111). It was found that activation of Hh signaling could mediate the interaction between cancer cells such as hepatocellular carcinoma cells and mammary carcinoma cells and macrophages and stimulate selective polarization of macrophages toward the M2 subtype, increasing the expression of pro-tumor factors such as TNF-α, iNOS and IL-6, and ultimately promoting tumor growth and metastasis (111, 112). Activation of M2 macrophages in the lung is regarded to be a crucial component in increasing lung fibrosis, and it was discovered that Shh increases the secretion of osteopontin in macrophages *via* the Shh/Gli signaling cascade, and the secreted osteopontin act on surrounding macrophages in an autocrine or paracrine manner and induce macrophage M2 polarization *via* the JAK2/STAT3 signaling pathway, which may ultimately promote the progression of pulmonary fibrosis by affecting the activation and proliferation of fibroblasts and the production of pro-fibrotic cytokines (113). Therefore, targeting the Shh/Gli/JAK2/STAT3 signaling pathway is beneficial for the treatment of macrophage-related diseases.

#### 4.3.5 Wnt signaling pathway

Typically, the Wnt signaling pathway is divided into two categories: typical Wnt/β-catenin signaling pathway and atypical Wnt signaling pathway, which are mainly activated by the 19 mammalian Wnt proteins (114, 115). STAT3 is one of the downstream targets of typical Wnt/β-catenin signaling. *In vitro*, Wnt3a, a molecule that promoted typical Wnt signaling pathway, exacerbated IL-4 or TGF-β1-induced macrophage M2 polarization as well as STAT3 phosphorylation and nuclear translocation; conversely, which in turn inhibited macrophage M2 polarization and suppressed the expression of the profibrotic cytokines PDGF, VEGF, TGF-β and connective tissue growth factor, thereby alleviating renal fibrosis (116). Similar studies have shown that β-catenin can regulate STAT pathway activation in macrophages and that ablation of β-catenin leads to STAT3 downregulation and STAT1 activation, resulting in elevated macrophage inflammatory responses and increased atherosclerosis (117, 118). The Ca(2+)/calmodulin-dependent kinase II (CaMKII) pathway is one of the important atypical pathways of Wnt5a signaling, which is required to mediate the Ca(2+)/CaKMII/STAT3 pathway in macrophage M2 polarization, which then promotes the expression of IL-10, TGF-β, CCL17, CCL18 and CCL22, thereby promoting proliferation, migration and invasion of rectal cancer cells. This signaling pathway may be a novel potential immunotherapeutic target against rectal cancer (119). Aberrant regulation of this pathway is closely associated with different diseases, suggesting that the Wnt/STAT3 signaling pathway is an attractive target for treatment of diseases.

#### 4.3.6 MAPK signaling pathway

The extracellular signal-regulated kinases 1 and 2 (ERK1/2), c-Jun amino-terminal kinases, p38, and ERK5 families of conventional MAPKs are the most well-known (120). The crosstalk between the p38 (MAPK) pathway and STAT3-mediated signaling is a fundamental axis of continuous LPS activation, and the balanced activation of this axis is essential to control the induction, propagation, and waning phase of the inflammatory macrophage response (121). In research on inflammatory lung disease, suppressing alveolar macrophage p38 (MAPK) expression almost entirely prevented STAT3 phosphorylation, thereby reducing the production of the inflammatory factor TNF-α, a cytokine known to be involved in the pathogenesis of inflammatory lung disease (122). Inhibition of this signaling pathway was shown to inhibit the polarization of pro-inflammatory M1-type macrophages and reduce the secretion of pro-inflammatory factors iNOS, IL-1, IL-6 and TNF-α, thereby alleviating adipose tissue inflammation (123). In addition to MAPK being associated with pro-inflammation, it was found that in ERK5-deficient macrophages, STAT3 phosphorylation process was blocked and the expression of pro-tumor factors (M2 factors) VEGF, IL-10 and TGF-β was reduced, which in turn prevented the growth of melanoma and lung cancer in mice (124). As a result, MAPK regulation of STAT3 phosphorylation can be utilized to affect the polarization of macrophages, which in turn can regulate the inflammatory immune response and inhibit the advancement of tumors.

In summary, multiple signaling pathways can regulate macrophage polarization by affecting STAT3, which in turn affects the development of disease. Therefore, activation or inhibition of these signaling pathways could regulate the M1/M2 ratio and provide new therapeutic strategies for related diseases (**Table 1**). However, the fact that the mechanisms involved in the regulation of macrophage polarization by STAT3 are extensive and complex in multiple diseases. This may be related to the heterogeneity of STAT3 and macrophages themselves, and the process involves the crosstalk of multiple signaling pathways, which may explain why STAT3 does not regulate macrophage polarization in the same direction in different diseases.

**Table 1:** Regulation of macrophage polarization by targeting STAT3 for the treatment of related diseases.

Disease classification	Disease	Experimental Model	Related signaling pathway	Impact on STAT3	Phenotype promoted	Affect	Reference
Inflammatory immunological disorder	Pancreatitis	Rat	JAK2/STAT3↓	↓	M2	Reduced pancreatic inflammation	(40)
	Myocardial ischaemia/ reperfusion injury	Rat	JAK2/STAT3↓	↓	M2	Inhibited cardiac apoptosis and reduced inflammation	(41)
	Psoriasis	Mouse	JAK/STAT3↓	↓	M2	Alleviated the hyperkeratotic epidermis and reduced inflammation	(42)
	Atopic dermatitis	Mouse	JAK2/STAT3↑	↑	M2	Ameliorated skin lesion and reduced inflammation	(44)
	Gouty arthritis	Rat	JAK2/STAT3↑	↑	M2	Reduced arthritis inflammation	(45)
	Non-alcoholic steatohepatitis (gut-liver axis)	Mouse	IL-10/(JAK)/STAT3↑	↑	M2	Alleviated barrier dysfunction and mitigated bacterial translocation	(58)
	Rheumatoid arthritis (RA)	Macrophage of RA patients	IL-10/(JAK1)/STAT3↑	↑	M2	Inhibited inflammatory cytokines and increased phagocytosis	(59)
	Nephritis	a. Rat’s bone marrow-derived macrophage b. Rat	SOCS3↓	↑	M2	Reduced inflammation	(66)
	Diabetic wounds	a. Human umbilical cord mesenchymal stromal cell and macrophage b. Rat	NF-κB↓ AKT↑	↑	M2	Reduced inflammation and promoted wound healing	(80)
Cancer	Colorectal cancer	a. Human colorectal cancer cell and macrophage b. Mouse	JAK2/STAT3↓	↓	M1	Inhibited colorectal cancer cells proliferation and migration Inhibited tumor growth and metastasis	(43)
			Wnt5a↓	↓	M1		(119)
	Gastric cancer	a. Human gastric cancer cell b. Mouse	IL-(6/8)/JAK2/STAT3↓	↓	M1	Inhibited gastric cancer cells migration, invasion and epithelial-mesenchymal transition Inhibited intratumor proliferation and angiogenesis	(55)
	Non-small-cell lung cancer (NSCLC)	a. Human NSCLC cell and macrophage b. Mouse	JAK(1/2)/STAT(3/6)↓	↓	M1	Inhibited NSCLC cells proliferation and migration Inhibited tumor growth and metastasis	(48)
	Ovarian Cancer	Human ovarian cancer cell and macrophage	IL-6/JAK2/STAT3↓	↓	M1	Inhibited IL-6 Secretion of ovarian cancer cells	(46)
	Gliomas	a. Human glioma cell and macrophage b. Mouse	IL-6/JAK2/STAT3↓	↓	M1	Inhibited tumor cells clone formation, migration, invasion and tube formation in vascular endothelial cells	(47, 49)
	Breast cancer	a. Human breast cancer cell and macrophage b. Mouse	SOCS3↑	↓	M1	Inhibited breast cancer cell invasion, the tumor growth and metastasis	(69)
			JAK2/STAT3/Notch↓	↓	M1		(109)
	Pancreatic adenocarcinoma	Mouse	NF-κB↓	↓	M1	Inhibited proangiogenic and tumor-promoting activity	(81)
	Liver cancer	a. Human liver cancer cell and macrophage b. Mouse	NF-κB↓	↓	M1	Inhibited the proliferative, invasive, and migratory ability of liver cancer	(86)
	Bladder cancer	a. Murine bladder cancer cell and macrophage b. Mouse	PI3K/AKT↓	↓	M1	Inhibited bladder tumor cell growth	(100)
	Lung carcinoma Melanoma	a. Murine cancer cell and macrophage b. Mouse	MAPK/ERK↓	↓	M1	Inhibited tumor cell proliferation and tumor angiogenesis	(124)
Fibrosis	Cardiac fibrosis	Rat	PI3K/AKT↑	↑	M2	Promoted wound healing and attenuated myofibroblast infiltration	(97)
	Pulmonary fibrosis	a. Murine lung ATII-like epithelial cell and macrophage b. Mouse	Shh/Gli↓	↓	M1	Inhibited pulmonary fibrosis	(113)
	Kidney Fibrosis	a. Murine macrophage b. Mouse	Wnt3a/β-catenin↓	↓	M1	Ameliorated kidney fibrosis	(116)

↓ means inhibition; ↑ means promotion.

## 5 Summary and prospect

Macrophages are highly plastic and can be polarized into M1 and M2 types under various environmental stimuli, and the functions of the two are almost antagonistic to each other. It is well known that macrophage polarization is a key to the pathogenic development of many disorders. As an extremely important transcription factor with multiple biological functions, STAT3 is the convergence point of several major signaling pathways involved in inflammatory, immunity, fibrosis, and oncogenic diseases, as well as a key regulator of macrophage different biological functions. STAT3 is a heterogeneous protein that can influence macrophage polarization in both directions, with the activation of it mostly regulating macrophage M2 polarization. However, there are no relevant studies to explain this paradoxical phenomenon, therefore, more exploration is needed to clarify the specific mechanism of STAT3’s role in macrophage polarization. The JAK/STAT signaling pathway is the most prominent pathway involved in STAT3 signaling, and the negative regulators SOCS, PIAS and PTP can affect macrophage polarization by inhibiting different targets of this signaling pathway, suggesting that targeting the STAT3 signaling pathway to alter the M1/M2 ratio appears to be effective in controlling disease progression. The current studies showed that the NF-κB signaling pathway, PI3K/AKT signaling pathway, Notch signaling pathway, Hedgehog signaling pathway, Wnt signaling pathway and MAPK signaling pathway crosstalk with JAK/STAT3 signaling pathway, which regulates gene transcription by affecting the phosphorylation and nuclear translocation of STAT3, have effects on macrophage polarization. This review summarized these signaling pathways with relevant diseases involved in this process to providing new ideas for the interested readers. However, most studies focused on single signaling pathway regulating macrophage M1 or M2 polarization, and very few studies investigated how other macrophage subtypes and multiple signaling pathways work together to regulate the direction of macrophage polarization and thus influence disease progression. Because of the diversity of STAT3 and macrophage activities, the complexity of signaling pathway transduction and regulation, and the network connectivity of these signaling pathways, the phenotypic alteration of macrophages is uncertain. The signaling pathways and regulatory mechanisms of STAT3 and macrophage polarization have not yet been thoroughly and systematically studied. In future work, the fundamental questions are whether there are new subtypes of STAT3 and what other important intermediate types of macrophages exist in addition to M1 and M2. The exploration of STAT3 and various subtypes of macrophages is the primary challenge. On this basis, more research should investigate how crosstalk of multiple signaling pathways affects the direction of macrophage polarization and how to achieve therapeutic goals by modulating macrophage phenotypic transition. These results will then be applied to study the pathogenesis of human-related diseases and explore more targeted treatment options. At present, most of the research is based on animal and cell experiments, and there is still a long way to go to translate them into clinical applications.

## Author contributions

TX wrote the original review and MZ, WL and TZ reviewed edited and assisted with figures. All authors contributed to the article and approved the submitted version.
